# Comparison of Effects of Pulsed Field Ablation and Cryoablation on Left Atrial Stiffness Using Invasively Derived Pressure–Volume Relationship

**DOI:** 10.1002/joa3.70520

**Published:** 2026-11-05

**Authors:** Christos‐Konstantinos Antoniou, Dimitris Tsiachris, Aikaterini‐Eleftheria Karanikola, Michail Botis, Athanasios Kordalis, Ioannis Doundoulakis, Panagiotis Xydis, Dimitrios Klettas, Christina Chrysohoou, Konstantinos P. Tsioufis

**Affiliations:** ^1^ First Department of Cardiology National and Kapodistrian University of Athens, “Hippokration” G.H.A. Athens Greece

**Keywords:** ablation, atrial fibrillation, atrial mechanics, pressure–volume relationship, pulsed field ablation

## Abstract

**Background:**

Acute effects of pulsed field ablation (PFA) on passive atrial mechanical properties have not hitherto been assessed by reconstruction of pressure–volume relationship using invasive intracavitary pressure measurements.

**Methods:**

In a cohort of 38 first‐time atrial fibrillation ablation patients, submitted to pulmonary vein isolation, we studied the effects of PFA and cryoballoon ablation on the left atrial elastic constant and passive elastic chamber stiffness constant, expressing baseline left atrial conditions and quantifying passive left atrial stiffness, respectively. Left atrial pressure was invasively measured immediately pre‐ and post‐procedurally, while volume values were obtained echocardiographically.

**Results:**

No differences were noted in passive elastic chamber stiffness constant values, pre‐ and post‐procedurally in either group. No differences were noted between CBA and PFA groups. There were no observable associations between Δ*α* and Δ*b* and either AF duration or preprocedural left atrial volume.

**Conclusions:**

Based on the above, CBA and PFA demonstrated comparable effects on left atrial mechanical properties in the acute post‐ablation setting.

## Graphical abstract

No differences were noted between pulsed field ablation and cryoballoon ablation regarding mechanical properties of the left atrium, assessed by invasively acquired *P*–*V* relationship, following first‐time ablation for atrial fibrillation.

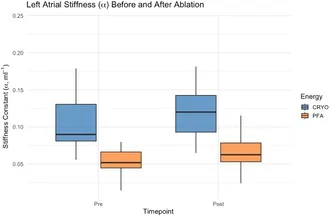

## 1 Introduction

Pulmonary vein isolation (PVI) currently constitutes the treatment of choice for atrial fibrillation (AF) [1]. Besides improved long‐term arrhythmia‐free survival, overall survival, and quality of life in selected populations, PVI may also lead to the occurrence of “stiff left atrium syndrome” associated with left atrial mechanical impairment due to extensive repair fibrosis, affecting its reservoir and booster pump functions [2]. Studies have reported increases in left atrial pressures following thermal energy‐based catheter ablation, with extension of energy application beyond pulmonary vein antra being one of the risk factors [2, 3, 4]. By using invasively acquired pressure volume loops, we have previously reported a slight but statistically significant difference between radiofrequency ablation and cryoablation regarding atrial stiffness immediately post‐procedurally [5].

The advent of pulsed (electrical) field ablation (PFA) has opened new possibilities regarding ablation targets and extent [6]. More specifically, the graded sensitivity of cell membranes of different tissues and the high sensitivity of myocardial cells' membranes to pore formation after electrical field application (electroporation) create a “myocardial selectivity” effect. Furthermore, the main mechanism of myocyte death is not necrosis, an inflammation‐associated and tissue structure‐disrupting process, but apoptosis, a form of programmed cell death without sequelae for neighboring structures and less scar formation [7]. The combination of the above allows for energy delivery without concern for affecting nearby structures or causing PV stenosis when energy is applied within sleeves [8].

However, PFA lesions exhibit a stacking effect [9], with additional application at the same site increasing effect and depth. Moreover, extensive ablation may lead to a condition where a less disruptive energy form is applied too extensively or vigorously, thus negating its advantage. Indeed, although initial cardiac magnetic resonance‐based assessments of residual fibrosis 3 months after PFA PVI showed no persistent late gadolinium enhancement [10], newer findings, albeit using a different PFA system, report “dense and homogeneous” fibrosis around PV antra [11].

Considering potential ramifications of the above regarding ablation technique, we attempted to assess left atrial passive mechanical properties in patients receiving first‐time PVI immediately after procedure completion, using invasively acquired pressure data, evaluating the effects of thermal and nonthermal energy on these parameters.

## 2 Patients and Methods

Thirty‐eight patients submitted to first‐time PVI were included. One group (*n* = 18) received cryoballoon ablation (CBA) using the POLARx Cryoballoon system and the other (*n* = 20) received PFA using the Farapulse system (both systems by Boston Scientific, Marlborough, MA, USA). Baseline characteristics for each group are shown in Table 1.

**TABLE 1:** Baseline characteristics of patients.

	CBA (*N* = 18)	PFA (*N* = 20)	*p*
Age (years)	62.5 (56.5, 68.0)	66.0 (60.3, 69.0)	0.35
Sex (Male)	11 (61%)	11 (55%)	0.7
BMI	27.1 (24.7, 30.2)	27.9 (26.1, 31.1)	0.81
CAD	2 (11%)	2 (10%)	0.91
HTN	9 (50%)	11 (55%)	0.76
HF	1 (5.6%)	2 (10%)	0.61
DM	3 (16.7%)	1 (5%)	0.24
CHA_2_DS_2_VA score	1.5 (0.2)	1.0 (0.3)	0.92
OSA	2 (11%)	2 (10%)	0.91
GERD	3 (16.7%)	2 (10%)	0.54
AF duration (years)	4 (1.13)	1 (1.5)	0.13
AF type, paroxysmal	15 (83%)	19 (95%)	0.24
AF type, persistent	3 (17%)	1 (5%)	0.24
AADs pretreatment	7 (50%)	13 (72%)	0.2
β‐blockers	14 (82%)	11 (69%)	0.36
Prior DCCV	5 (83%)	11 (92%)	0.6
Anticoagulation	15 (88%)	18 (90%)	0.86
LVEF (%)	60 (54, 60)	60 (60, 60)	0.06
LA Diameter (mm)	39 (37, 45)	42 (39, 46)	0.08
**LA ESV (mL)**	**54 (46, 63)**	**68 (56, 86)**	**0.007**
**LAVi**	**28 (23, 31)**	**39 (27, 44)**	**0.01**
Procedure duration (min)	70 (60, 75)	60 (48.8, 80)	0.36
LA time (min)	45 (30, 50)	37.5 (30, 52.5)	0.91

*Note:* Values in *n* (%) for nonparametric variables and in mean (25th–75th quartiles) for parametric ones. Bold value denotes parameters with statistically significant differences between ablation groups.

Abbreviations: AAD, antiarrhythmic drug; AF, atrial fibrillation; BMI, Body mass index; CAD, coronary artery disease; CBA, cryoballoon ablation; DCCC, direct current cardioversion; DM, diabetes mellitus; ESV, end‐systolic volume; GERD, gastroesophageal reflux disease; HTN, hypertension; LA, left atrium; LAVi, left atrial volume index; LVEF, left ventricular ejection fraction; OSA, obstructive sleep apnea; PFA, pulsed field ablation.

In both groups, left atrial pressure was measured invasively, through the FARADRIVE steerable sheath, immediately after transseptal puncture and after completion of lesion formation, just prior to retraction of the sheath to the right atrium. Fluoroscopic navigation was used in all cases. Left atrial volume values during the passive filling phase were acquired through echocardiography immediately prior to the procedure. More specifically, following closure of the mitral valve, LA pressure changes were monitored through the sheath, and the minimum value was recorded and projected to the ECG tracing. Thus, the corresponding volume of the atrium at the beginning of the clockwise ascending limb of the PV loop (representing pressure–volume relationship during passive filling of the atrium) was measured and the first pair of values was acquired. When the pressure reached the inflection point, again through the ECG tracing, the corresponding atrial volume was recorded, and the second point acquired. Further pressure‐volume value pairs were acquired at regular time intervals between these 2 points. As previously described [5], data from the experimental curve were then fitted to the equation $P=be^{\mathrm{aV}}$, where *P* and *V* were the experimental values of pressure and volume, respectively, *b* represents the elastic constant (in mmHg), expressing baseline left atrial conditions, and *α* is the passive elastic chamber stiffness constant (in mL^−1^), quantifying passive left atrial stiffness—see Figure 1 for a graphic representation. In accordance with previous practice, at least 12 pressure‐volume value pairs were used to construct the experimental curve.

**FIGURE 1:**
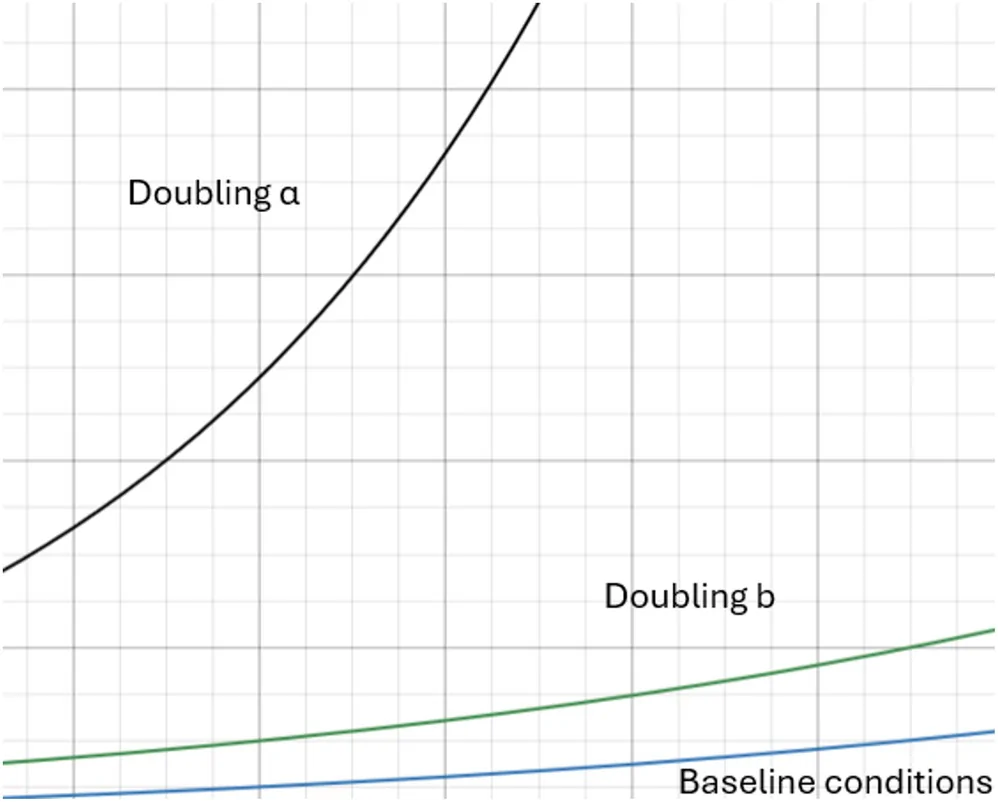
Comparative effects of similar changes in *α* and *b* parameters (doubling) on the ascending limb of the *P* = *f*(*V*) relationship. Effects of changes in *α* greatly outweigh effects of changes in *b*.

We focused on the differences (Δ) in each parameter (*α* and *b*) within each energy group pre‐ and post‐procedurally as a means to account for differences in atrial size and given the nonrandomized nature of the study.

## 3 Statistical Analysis

Continuous variables are presented as median and interquartile range (IQR). Within each treatment group, pre‐ and post‐ablation values of the stiffness constant (*α*) and intercept (*b*) were compared using the paired Wilcoxon signed‐rank test. The associations of pre‐ablation *α*/Δ*α* and *b*/Δ*b* with maximum atrial fibrillation duration were assessed using Pearson's correlation coefficient. A two‐sided *p* value < 0.05 was considered statistically significant.

## 4 Results

Baseline characteristics did not differ significantly between ablation groups, with the exception of left atrial size, which was larger in the PFA group.

Concerning left atrial mechanical properties (Table 2), prior to ablation, there were no differences between groups. Post‐lesion administration, in the CBA group, there was a significant reduction in the elastic constant (*b*). Neither *α* in the CBA group, nor *α* and *b* in the PFA group exhibited any statistically significant difference per‐ and post‐procedurally. Results concerning *α* are visualized in Figure 1.

**TABLE 2:** Results regarding left atrial passive mechanical properties.

	CBA‐pre	CBA‐post	ΔCBA (median of all intra‐patient differences)	*p* (pre‐ vs. post‐)
*α* (mL^−1^)	8.98 × 10^−2^ (4.95 × 10^−2^)	12 × 10^−2^ (4.95 × 10^−2^)	1.32 × 10^−2^	0.204
*b* (mmHg)	7.42 × 10^−2^ (24 × 10^−2^)	3.51 × 10^−2^ (6.90 × 10^−2^)	−6.38 × 10^−2^	**0.034**

	PFA‐pre	PFA‐post	ΔPFA (median of all intra‐patient differences)	*p* (pre‐ vs. post‐)
*α* (mL^−1^)	5.19 × 10^−2^ (2.16 × 10^−2^)	6.25 × 10^−2^ (2.54 × 10^−2^)	0.286 × 10^−2^	0.210
*b* (mmHg)	36.4 × 10^−2^ (45.4 × 10^−2^)	21.6 × 10^−2^ (42.8 × 10^−2^)	0.622 × 10^−2^	0.709

*Note:* Results as mean (SD). Bold value denotes parameters with statistically significant differences between ablation groups.

Abbreviations: CBA, cryoballoon ablation; PFA, pulsed field ablation.

Despite the nonrandomized nature of our data, we performed an exploratory analysis of differences in Δ*α* and Δ*b* between groups, using Wilcoxon's test, which yielded statistically nonsignificant differences (*p* = 0.29 and *p* = 0.11 for Δ*α* and Δ*b* between groups, respectively—Figure 2).

**FIGURE 2:**
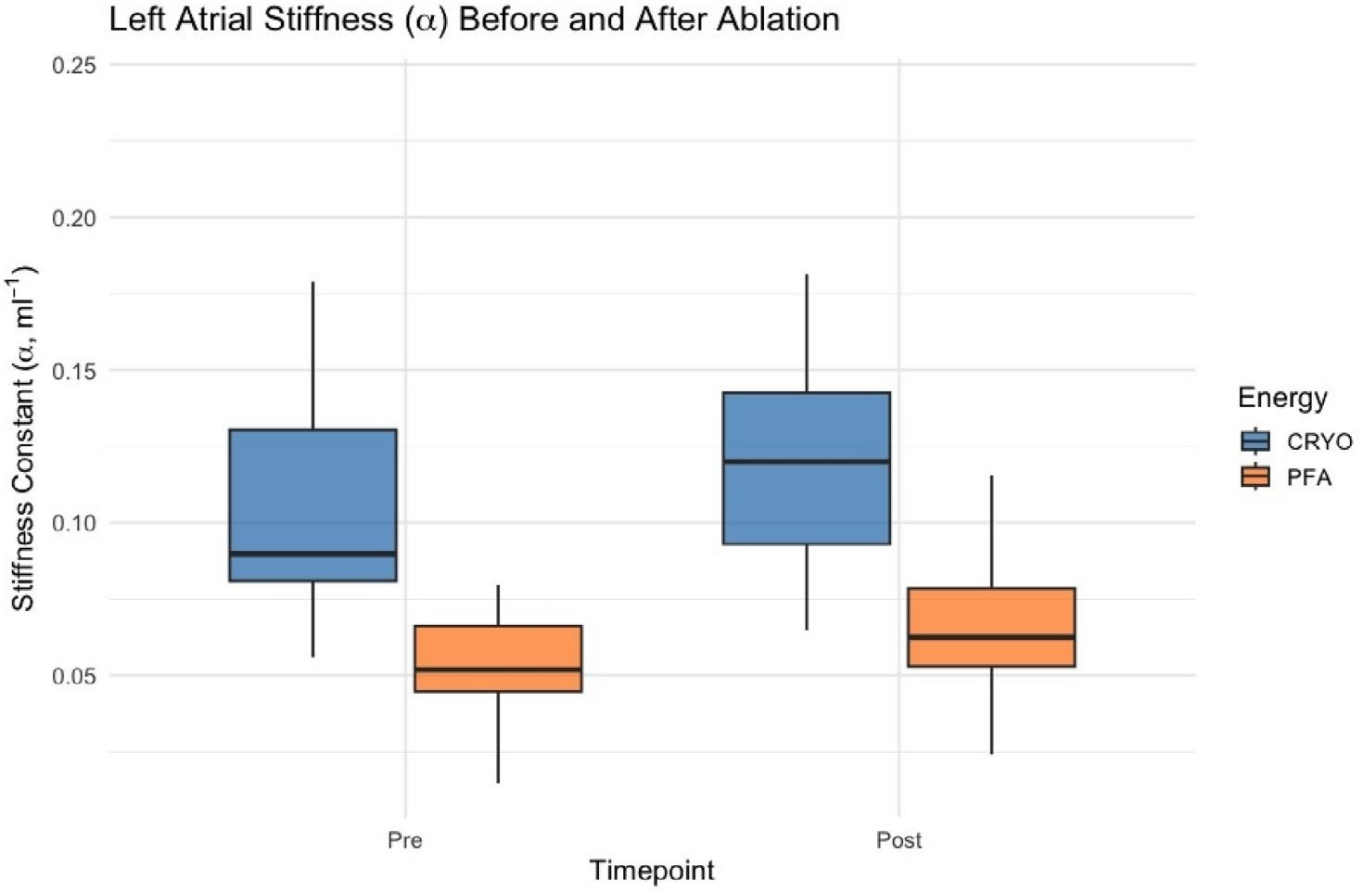
Comparison of *α* values pre‐ and post‐procedurally, per energy type.

No differences regarding left atrial dimensions were noted at the 3‐month follow‐up (Table 3).

**TABLE 3:** Differences in left atrial dimensions at 3‐months follow up.

	Group	*p*
LA end systolic volume	CBA	0.76
	PFA	0.87
LA volume index	CBA	0.28
	PFA	0.87
LA diameter	CBA	0.27
	PFA	0.27

Abbreviations: CBA, cryoballoon ablation; LA, left atrium; PFA, pulsed field ablation.

No association was found between presenting LA volume and either Δ*α* or Δ*b*. This holds true for the overall cohort, as well as for either energy type group (*p* = 0.195 for Δ*α*, and *p* = 0.227 for Δ*b* in the overall cohort).

Finally, regarding the relationship between AF duration and LA mechanical properties, time data were available for 24 patients. There was no statistically significant correlation between AF duration and any of *α*, *b*, Δ*α*, and Δ*b* (data for *α* and *b* shown in Figure 3).

**FIGURE 3:**
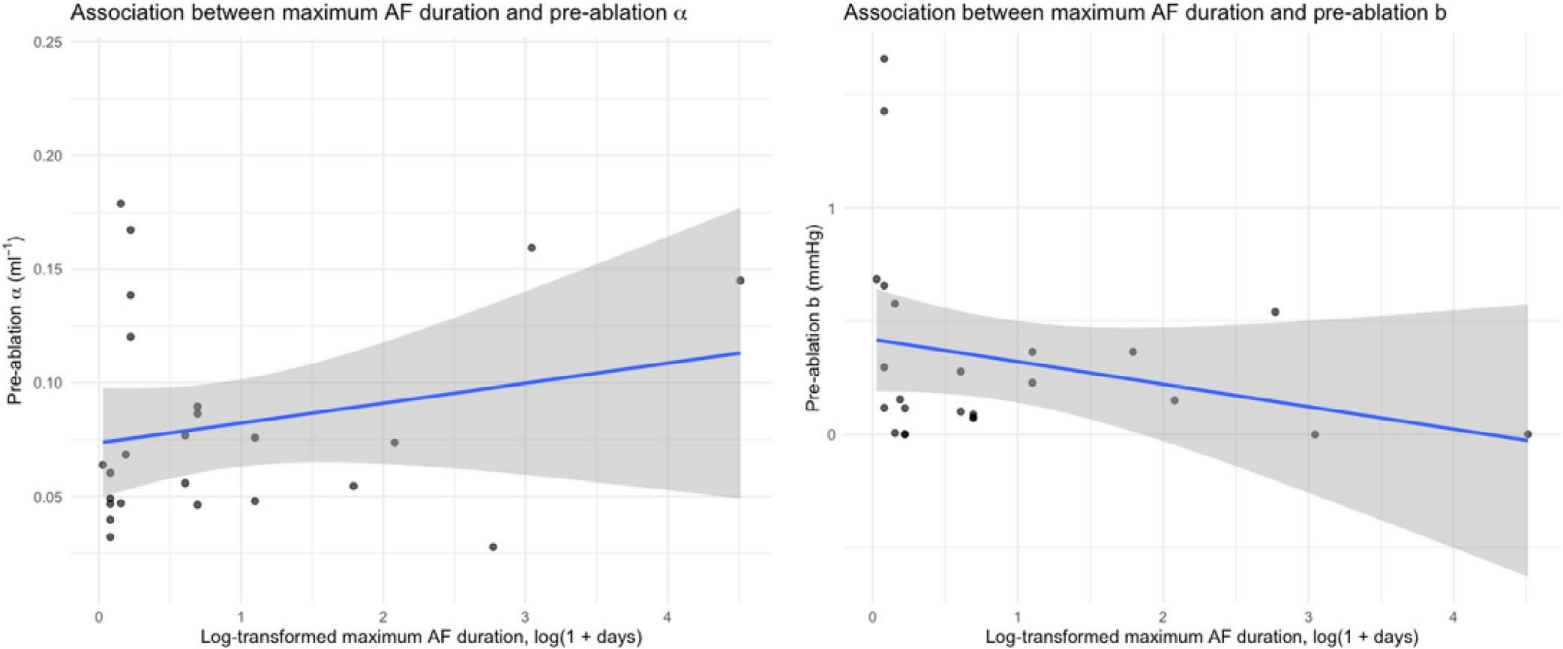
(Lack of) correlation between max AF duration and baseline *α*/*b*.

## 5 Discussion

Based on our findings, both CBA and PFA demonstrated comparable effects on left atrial mechanical properties in the acute post‐ablation setting, with no significant changes in the passive stiffness constant (*α*) after either procedure. The mild reduction in the elastic constant (*b*) with CBA did not alter overall atrial elasticity (expressed by *α*), suggesting that both modalities preserve left atrial compliance acutely.

To the best of our knowledge, our work is the first to have used invasive pressure measurements to assess the effects on left atrial passive mechanical properties of CBA and PFA, using a sample size similar to a previous comparison between RFA and CBA [5]. Previously, a small study reported a trend toward maintained left atrial volumes with PFA, as opposed to decreased with RFA, in cases where extensive ablation was performed (PVI, posterior wall isolation and left isthmus), implying no scar‐related volume contraction of the left atrium [12]. However, this study only relied on volumetric data, forsaking pressure data which would have allowed for direct estimates of left atrial compliance/stiffness and would have clarified the meaning of its findings—that is, volume reduction in RFA with reduced stiffness would have been due to reverse atrial remodeling (positive) whereas volume reduction with increased stiffness would imply a shriveled, fibrotic atrium (negative). Another study compared left atrial mechanical function, based on echocardiographically‐derived 2‐dimensional strain, following PFA and RFA and concluded that the former was associated with similar outcomes regarding reservoir function but *worse* regarding atrial contractile function at 3 months [13]. Authors surmise that preservation of extracellular matrix structure with PFA could interpret the normalization of passive left atrial properties at 3 months, whereas damage to atrial conduction system (Bachmann bundle), shown to occur with PFA [13, 14, 15] due to increased transmurality/broader area of application, could underlie impaired contractility.

Our work aimed to assess the effects of CBA and PFA in first‐procedure PVI. It must be clarified that the design of the study does not allow for assessing acute changes in contractility, given the focus on the *P*–*V* relationship during passive atrial filling. Consequently, we attempted to evaluate acute ablation effects on passive mechanical atrial properties, mainly determined by changes in extracellular tissue architecture. Such changes may stem from either acute inflammation and swelling (thermal‐based energy, in this case CBA) or from the release of mediators by reversibly electroporated/stunned myocardial cells (PFA).

Left atrial size was found to be significantly higher in the PFA group. Inasmuch as this was not a randomized trial, it could be attributed to a tendency of using PFA in cases of more dilated left atria, given its larger area footprint and the possibility of not successfully occluding larger veins with a cryoballoon. Such atrial usually, but not always, have undergone remodeling and thus have stiffer architecture, that is, increased *α* values—indeed that was the trend we observed. Given this, the fact that Δ*α* did not differ between energy‐based groups in our *exploratory* analysis, and that *stiffer atria stiffen more easily* [16, 17], PFA would appear to compare not unfavorably to CBA, even when applied to a less ideal substrate. On the other hand, it should be highlighted that oftentimes dilated atrial are found to exhibit normal voltage values in substrate electroanatomical mapping during ablation, that is, being not overly fibrotic—consequently the importance of magnetic resonance/substrate mapping data becomes apparent.

The type of energy applied significantly affected only the value of *b* (elastic constant) in the CBA group, where a significant reduction was noted, as opposed to a nonsignificant increase in *b* in the PFA group. The larger atria in PFA patients, based on Laplace's law, operate at higher pressures, reflected in the higher value of *b*, expressing baseline operating conditions. Furthermore, it could be hypothesized that the smaller diameter of sheaths used in CBA led to lower rates of saline perfusion to maintain patency, prevent thromboembolism, and mitigate air intrusion, leading to lower baseline pressures.

Concerning *α* (passive elastic chamber stiffness constant), no significant differences were noted in either group, in the acute post‐ablation setting. Notably, CBA outperformed radiofrequency ablation regarding effects on *α* and *b* in a similar study [5]. Obviously, the transitive property cannot be applied, yet our results are somewhat reassuring concerning the use of PFA and its acute effects on passive mechanical properties of the atrium (expressed mainly by *α*). Baseline atrial conditions are affected by hydration status, infusion, and ventricular rates, in turn affected by sedation depth. Moreover, PFA methodology has not yet been streamlined and a host of parameters, including catheter design and footprint, field properties, and application train features may affect induced changes in atrial mechanics.

AF ablation has a pivotal role in the management of heart failure, irrespectively of ejection fraction [18, 19, 20]. Given the increased tendency of atrial fibrotic remodeling in HFpEF [21] and the beneficial effects of (thermal) ablation on atrial parameters [22], studying the effects of PFA on atrial mechanics becomes even more attractive.

PFA is a relatively new approach to cardiac ablation. A method with (mostly) nonthermal effects that has nevertheless begun to compare favorably to established modalities, such as CBA [23]. Fine tuning of PFA is still underway, including accurate assessment of stacking effect, calculation of lesion depth, optimal electrical field shape and pulse duration (nsec vs. μsec) [24, 25]. To add to all this, the complexity of the underlying atrial mechanics is evidenced by the fact that, despite left atrial size being larger in the PFA group, preprocedural *α* values were similar, as well as by the lack of association between initial LA volume and subsequent changes in *α* and *b* (Δ*α* and Δ*b*).

The lack of association between AF duration and mechanical parameters is also supportive of the notion of differing atrial remodeling among patients (e.g., related to proinflammatory/profibrotic tendency, or comorbidities, such as diabetes and hypertension). Thus, histological factors could be relevant, rendering magnetic resonance imaging, or data from invasive electroanatomical mapping, useful for more accurate assessment.

On a more fundamental level and given that membrane electroporation is not the sole cause for cellular death with PFA, more issues arise, including whether successive applications at the same site may convert reversibly electroporated tissues to irreversibly electroporated and whether this may also apply to tissues theoretically insensitive to the electrical field intensity used for myocyte ablation.

## 6 Limitations

The small, nonrandomized sample limits the robustness of comparisons and precludes between‐group comparisons, save for merely exploratory ones. Besides the above, magnetic resonance imaging was not used to assess and correlate occurrence of fibrosis in the chronic setting with acute changes in mechanical atrial properties. Furthermore, an invasive reassessment would be desirable; however, both logistical and ethical considerations would arise, especially in patients without arrhythmia recurrence.

## Funding

The authors have nothing to report.

## Conflicts of Interest

The authors declare no conflicts of interest.

## Data Availability

The data that support the findings of this study are available on request from the corresponding author. The data are not publicly available due to privacy or ethical restrictions.
